# Hospital Meetings

**Published:** 1915-12-04

**Authors:** 


### Hospital Meetings.

### MIDDLESEX HOSPITAL.

A MEETING of the Court of Governors of Middlesex Hospital was held on Thursday, November 25, Mr. Arthur James, vice-president of the hospital, in the chair.

Among the items of the subscription list the effort of La Fraternité Masonic Lodge, which is mainly composed of foreign members, is worthy of special note. About three years ago the Lodge asked whether the hospital authorities would allow them to collect waste tin-foil from confectioners' and tobacconists' shops and receive for the hospital the proceeds of sales of such material. Much of the collecting was done by children, and it commenced in 1913. The total now contributed from that source was £327.

Since the commencement of the war 2,839 wounded soldiers have received treatment in the wards of the hospital or at the branch hospital at Clacton-on-Sea.

